# Very low levels of direct additive genetic variance in fitness and fitness components in a red squirrel population

**DOI:** 10.1002/ece3.982

**Published:** 2014-04-11

**Authors:** S Eryn McFarlane, Jamieson C Gorrell, David W Coltman, Murray M Humphries, Stan Boutin, Andrew G McAdam

**Affiliations:** 1 Department of Integrative Biology, University of GuelphGuelph, ON, N1G 2W1, Canada; 2 Department of Biological Sciences, University of AlbertaEdmonton, AB, T6G 2E9, Canada; 3 Department of Natural Resource Sciences, McGill University, Macdonald CampusSte-Anne-de-Bellevue, QC, H9X 3V9, Canada

**Keywords:** Genetic covariance, heritability, Robertson–Price identity, sexual antagonism, temporal fluctuations in selection

## Abstract

A trait must genetically correlate with fitness in order to evolve in response to natural selection, but theory suggests that strong directional selection should erode additive genetic variance in fitness and limit future evolutionary potential. Balancing selection has been proposed as a mechanism that could maintain genetic variance if fitness components trade off with one another and has been invoked to account for empirical observations of higher levels of additive genetic variance in fitness components than would be expected from mutation–selection balance. Here, we used a long-term study of an individually marked population of North American red squirrels (*Tamiasciurus hudsonicus*) to look for evidence of (1) additive genetic variance in lifetime reproductive success and (2) fitness trade-offs between fitness components, such as male and female fitness or fitness in high- and low-resource environments. “Animal model” analyses of a multigenerational pedigree revealed modest maternal effects on fitness, but very low levels of additive genetic variance in lifetime reproductive success overall as well as fitness measures within each sex and environment. It therefore appears that there are very low levels of direct genetic variance in fitness and fitness components in red squirrels to facilitate contemporary adaptation in this population.

## Introduction

The Robertson–Price identity states that a trait must be genetically correlated with fitness to evolve in response to natural selection (Robertson 1966; Price 1970, 1972). Both fitness and the trait in question must therefore have some level of additive genetic variance to be correlated (Houle 1991). As such, additive genetic variance in fitness is a prerequisite for evolution and provides an overall measure of the current adaptive capability of a population (Shaw and Shaw 2013), but it is rarely measured directly in studies of microevolution in the wild (Morrissey et al. 2010).

Fitness is a measure of the extent to which individuals contribute genes to the next generation through the processes of survival and reproduction (Brommer et al. 2002). Despite being a fundamental evolutionary concept, fitness is difficult to measure in a consistent way (Krimbas 2004), so several proxies have been used in empirical studies in lieu of true fitness. When individuals can be followed throughout their lifetime, their fitness can be determined as their lifetime reproductive success (LRS). LRS can be considered an individual's contribution to the net reproductive rate of a population (*R*_0_: Brommer et al. 2002). LRS does not account for reproductive timing (Brommer et al. 2002), so it might overestimate the relative fitness of females who time their reproduction later in life (Brommer et al. 2002), but it is intuitive, relatively easy to estimate, and is widely used as a measure of fitness in studies of microevolution in the wild (e.g., Gustafsson 1986; Kruuk et al. 2000; Merilä and Sheldon 2000).

Standing levels of genetic variation in any trait reflect a balance between mutation, which generates genetic variation, and selection, which often erodes genetic variation (i.e., mutation-selection balance; Barton and Keightley 2002). Fitness, however, is predicted to have little or no additive genetic variance because fitness is, by definition, under strong directional selection, and genes that consistently enhance fitness should quickly go to fixation (Robertson 1955; Fisher 1958; Jones 1987). This fundamental prediction of evolutionary theory, however, is inconsistent with empirical observations suggesting that levels of additive genetic variance of life history traits can be greater than additive genetic variance estimated for morphological or physiological traits (Houle 1992). Estimates of the heritability of fitness from wild populations range widely (Table 1), but in some cases, the coefficient of additive genetic variance (CV_a_) is surprisingly high, leading to an expectation of high evolutionary potential of fitness in these systems (Houle 1992; Shaw and Shaw 2013). For example, moderate CV_a_s were reported (percentiles were calculated based on all CV_a_ values for all traits reviewed by Hansen et al. 2011) for fitness in female collared flycatchers (29th percentile, Merilä and Sheldon 2000) and in male red deer (78th percentile, Foerster et al. 2007). The presence of moderate levels of additive genetic variance in fitness in the wild suggests the importance of some mechanism that maintains additive genetic variance in fitness or in fitness components in natural systems.

**Table 1:** Previous estimates of heritability and maternal effects of fitness in wild populations. Different fitness measures are denoted as lifetime reproductive success (LRS), delifing (Coulson et al. 2006) or relative LRS (LRS/meanLRS)

Study	Study organism	Heritability estimate	Maternal effects estimate	Fitness measure
Gustafsson (1986)	Female collared flycatchers	0.01 ± 0.16	n/a	LRS
Gustafsson (1986)	Male collared flycatchers	0.01 ± 0.13	n/a	LRS
Kruuk et al. (2000)	Female red deer	0.00 ± 0.05	0.16 ± 0.041	LRS
Kruuk et al. (2000)	Male red deer	0.02 ± 0.06	NS	LRS
Merilä and Sheldon (2000)	Female collared flycatchers	0.21 ± 0.06	n/a	LRS
Merilä and Sheldon (2000)	Male collared flycatchers	0.07 ± 0.06	n/a	LRS
McCleery et al. (2004)	Female great tits	0.00 ± 0.04	n/a	LRS
McCleery et al. (2004)	Male great tits	0.02 ± 0.04	n/a	LRS
Foerster et al. (2007)	Female red deer	0.09 ± 0.02	0.0021	Delifing
Foerster et al. (2007)	Male red deer	0.04 ± 0.03	0.0045	Delifing
Teplitsky et al. (2009)	Female red billed gulls	0.36 ± 0.29	NS	LRS
Teplitsky et al. (2009)	Male red billed gulls	0.00 ± 0.00	NS	LRS
Schroeder et al. (2011)	House sparrows	0.09 (0.03–0.18)	0.33 (0.14–0.51)	Delifing
Morrissey et al. (2012)	Soay sheep	0.0259 ± 0.0145	NS	Relative LRS

n/a indicates that maternal effects were not estimated in this study; NS indicates that maternal effects parameter was dropped from the model, based on an AIC model assessment.

Balancing selection is commonly proposed as a mechanism that can maintain genetic variation through trade-offs between fitness components (Barton and Keightley 2002). When trade-offs between fitness components exist, higher levels of genetic variation can be maintained in the components of fitness that trade off than total fitness (Mousseau and Roff 1987). Sexual antagonism is one form of balancing selection in which there is opposing selective pressures on males and females (Chippindale et al. 2001). Sexual antagonism occurs when selection in one sex moves the other sex away from its phenotypic optimum (Bonduriansky and Chenoweth 2009) and arises when there is sexual conflict over optimal values of a trait or a gene that is shared by females and males. Fitness trade-offs associated with sexual antagonism therefore represent a form of balancing selection and can maintain additive genetic variance in male and female fitness (Turelli and Barton 2004). For example, sexual antagonism in red deer, in which males with high fitness produced daughters with low fitness, was associated with high levels of genetic variance in male and female fitness (Foerster et al. 2007).

Temporal fluctuations in selection, where environmental changes lead to fitness trade-offs between time periods, have been suggested as a mechanism that could also maintain additive genetic variance (Levins 1968; Hedrick et al. 1976). Models have shown that genetic variance can be maintained via a storage effect if selection is intense, acts particularly on early life history stages, and there is a high level of generation overlap (Ellner and Hairston 1994). This is especially true when adults are relatively long lived and sheltered from episodes of selection (Ellner and Hairston 1994). For example, Turelli et al. (2001) found that temporal fluctuations in selection maintained color polymorphism in *Linanthus parryae,* where white flowers have a high relative fitness in years of high rain, which compensates for low relative fitness in other years (Turelli et al. 2001). This variation in relative fitness, in addition to a persistent seed bank, allowed for maintenance of genetic polymorphism (Turelli et al. 2001). Temporal fluctuations, however, have not yet been tested as a mechanism maintaining additive genetic variance in fitness in different environments in a wild population (Kruuk et al. 2008).

North American red squirrels (*Tamiasciurus hudsonicus*) are a good system in which to examine both sexual antagonism and temporal fluctuations in selection as forms of balancing selection that could maintain genetic variance in components of fitness. Red squirrels are diurnal, semi-arboreal rodents that are common throughout most of forested North America. Offspring depend on their mothers for nutrition prior to weaning at about 70 days after birth, while fathers provide no parental care (McAdam et al. 2002; Lane et al. 2007). Sexual conflict between parents, if genetically based, could lead to sexual antagonism in fitness. For example, female and male red squirrels were found to allocate time differently while hoarding, which suggests the potential for previous or ongoing sexual conflict (Archibald et al. 2013) Fig. 1.

**Figure 1:**
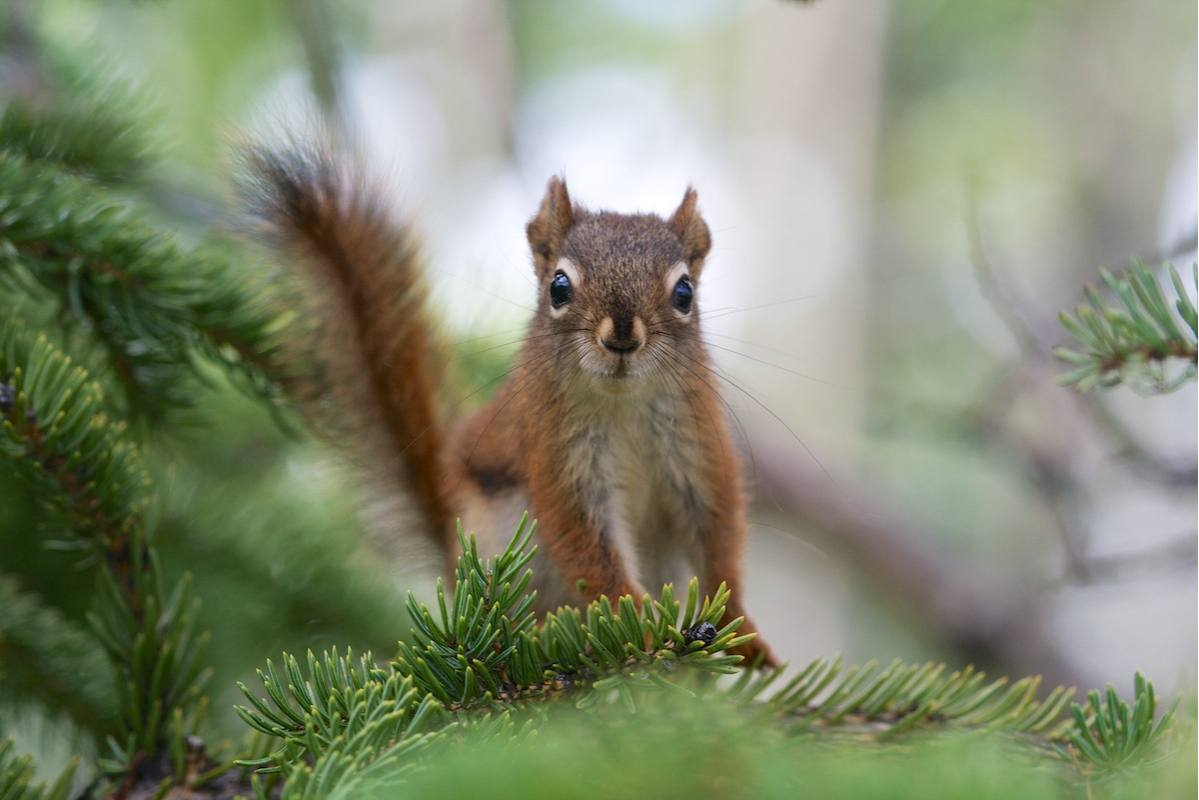
North American red squirrel (*Tamiasciurus hudsonicus*). Photo by Ryan W. Taylor.

In the southwest Yukon, red squirrels are territorial and depend on the seeds from white spruce cones (*Picea glauca*) as an important food source (Fletcher et al. 2010, 2013). Individuals store cones in a larder hoard called a midden and are dependent on their cache of spruce cones to survive the winter (Larsen and Boutin 1994). Juvenile red squirrels must obtain a midden and cache cones in order to survive the winter, so juvenile settlement on a territory represents a strong episode of selection in the red squirrel population (McAdam and Boutin 2003; McAdam et al. 2007). Every 3–6 years, white spruce trees synchronously produce large numbers of cones, which is a process called masting (LaMontagne and Boutin 2007). During mast years, spruce trees each produce many cones that saturate the ability of red squirrels to harvest cones (Fletcher et al. 2010). Mast years, in which food resources are superabundant, are typically followed by several years of no cone production or very low cone production (LaMontagne and Boutin 2007). These large fluctuations in food resources affect the population density of squirrels, the timing and amount of reproduction, and the strength of natural selection on offspring growth rates (Boutin et al. 2006; Dantzer et al. 2013). These resource pulses therefore present an opportunity to determine whether temporal environmental fluctuations could maintain additive genetic variance in fitness.

Here, we estimated the heritability of fitness in a natural population to try and determine the population's adaptive potential. We also assessed whether components of fitness had higher levels of additive genetic variation as would be expected if fitness trade-offs maintained variation in fitness components. Specifically, we assessed whether sexual antagonism maintained additive genetic variance in the fitness of male and female red squirrels by estimating the additive genetic variance in fitness for each sex separately and testing for a negative genetic correlation between male and female fitness, which would represent a fitness trade-off (Foerster et al. 2007). Similarly, we assessed whether a fitness trade-off between high- and low-resource conditions associated with white spruce masting maintained genetic variation in environmentally dependent fitness in red squirrels, by estimating the additive genetic variance in fitness within mast years and within nonmast years separately (sensu Vehviläinen et al. 2008) and testing for a negative genetic correlation between fitness in mast and nonmast environments.

## Materials and Methods

A natural population of North American red squirrels in the southwest Yukon, Canada (61°N, 138°W), was monitored from 1987 to 2011. The population was completely enumerated through regular livetrapping of individuals with unique alphanumeric ear tags (McAdam et al. 2007). To collect data on reproduction, squirrels in the population were routinely captured using Tomahawk live traps (Tomahawk Live Trap, Tomahawk, WI) and assessed for reproductive status. Females were regularly monitored for pregnancy status through abdominal palpation, assessment of nipple condition, and recorded weight. Once pregnancy was confirmed via weight loss or lactation, radio telemetry was used to locate nests. Pups were temporarily removed from the nest soon after birth to assess litter size and again at ∼25 days of age in order to collect DNA for paternity and permanently tag the pups (see McAdam et al. 2007). Permanently tagging pups within their natal nest allowed us to assign maternity with certainty. Paternity was assigned starting in 2003 using tissue samples taken from pups and adult immigrants that were genotyped at 16 microsatellites (Gunn et al. 2005) and analyzed with 99% confidence using CERVUS 3.0 (Kalinowski et al. 2007).

### Fitness Analysis

We used lifetime reproductive success (LRS; i.e., the total number of offspring that an individual produced during their lifetime) as our measure of fitness. Individuals were considered to be dead if they were not trapped for more than one year or if they were found dead (Descamps et al. 2009). All individuals who were included in our analyses died of natural causes. Adult squirrels in this population live an average of 3.5 years (McAdam et al. 2007). Survival does not appear to be confounded by dispersal in our system; we have completely enumerated the population via frequent livetrapping (McAdam et al. 2007), juveniles typically disperse <100 m (Larsen and Boutin 1994; Stuart-Smith and Boutin 1995; Berteaux and Boutin 2000; Kerr et al. 2007), the areas that surrounded our study areas were poor-quality squirrel habitat, and there was no evidence that individuals from the center of the study site were surviving better than those from the edge, as would be expected if individuals were successfully dispersing out of the study area (McAdam et al. 2007). Here, we assigned the early survival of pups to the fitness of the offspring and not the mother such that LRS was the total number of pups produced. This is in contrast to other studies (Foerster et al. 2007), which assigned the early survival of offspring to the fitness of their parent. We assigned early survival to the fitness of the offspring and not their parent (Wolf and Wade 2001) to avoid confounding fitness and inheritance, although we recognize that this assignment of early survival to offspring fitness is not always useful as it can result in biased phenotypic selection estimates due to selection acting on adult traits prior to their phenotypic expression (i.e., the invisible fraction; Grafen 1988). Nevertheless, analyzing fitness as the total number of recruited offspring did not affect our conclusions (results not shown). In addition to LRS, we also used annual reproductive success (ARS) as a proxy of fitness, which was analyzed as the number of pups produced in a year.

All individuals included in the analysis of LRS died before the 2011 breeding season to ensure that we had full data for their lifetime. Not all squirrels in our ARS models were deceased at the time of this analysis. We included 2981 individuals in our analysis of LRS and 1682 individual–year combinations in our analysis of ARS. We began estimating paternity in 2003, so we estimated the LRS of males beginning with males born in 2002. Older males were included in male ARS estimates, which began in 2003. Individuals included in the ARS analyses were all old enough to have bred when measured (i.e., 1 year or older). Only individuals with known mothers were included in our analysis, as we knew from previous work in this system that mothers could have large effects on fitness-related traits in their offspring (McAdam et al. 2002; Dantzer et al. 2013), and thus wanted to measure maternal effects on fitness (see below).

### Animal model analyses

Mixed-effect “animal models” combine phenotypic information on individuals with information from a pedigree to determine how much related individuals resemble each other in order to estimate sources of variation including additive genetic variance (Kruuk 2004). The pedigree that we used included both paternal and maternal linkages and consisted of 7799 individuals, of which 6196 had known mothers and 1827 had known fathers. We used “animal models” to estimate variance components of additive genetic (*V*_a_), maternal (*V*_m_), and permanent environmental (*V*_i_) effects for all models of annual reproductive success and *V*_a_ and *V*_m_ for models of lifetime reproductive success (Kruuk 2004). Maternal effects in this case represent a consistent effect of a mother on the fitness of her offspring above and beyond her direct genetic contribution through the inheritance of offspring traits (Mousseau and Fox 1998). Permanent environmental effects represent the among-individual variance in fitness not explained by additive genetic variance (Kruuk and Hadfield 2007). This permanent environmental effect can only be estimated in traits that are measured for each individual more than once (Kruuk and Hadfield 2007) and hence cannot be estimated for LRS.

We used bivariate models to estimate genetic correlations between traits. To test the sexual antagonism hypothesis, we estimated genetic correlations between male and female LRS and between male and female ARS. To test the temporal fluctuations hypothesis, we estimated genetic correlations of both response variables in different environments: ARS in mast and nonmast environments and LRS of individuals born in mast and those born in nonmast environments. While individuals experience a range of environments during their lifetime, survival to one year of age represents a large component of lifetime fitness (McAdam et al. 2007), so the environment experienced during the first year of life might have important consequences for lifetime fitness. We used a character state approach, where we assumed environments to be binary, rather than random regressions because mast and nonmast years are dramatically different in resource levels (LaMontagne and Boutin 2007).

We estimated genetic correlations between traits even when univariate models indicated nonsignificant additive genetic variance in one or both of the traits. Logically, it does not make sense to estimate a correlation when one or both traits have no variance (Houle 1991), but our predictions were bivariate in nature, and bivariate models can sometimes provide more power to detect variances than univariate models, especially when traits are measured in different individuals (e.g., Svensson et al. 2009). That said, we expected large confidence intervals for correlations when one or both traits have low variance.

All models were run in R using the Markov Chain Monte Carlo for generalized linear mixed models (MCMCglmm) package (Hadfield 2010; R Development Core Team 2011). We assumed a Poisson distribution for all traits. The pedigree was modified to include only informative individuals using the “pedantics” package (Morrissey and Wilson 2010). Variance components were estimated as the mode from posterior distributions. The residual variance (*V*_r_) was estimated for LRS and ARS. Our bivariate models allowed for covariance between additive genetic variances and maternal effects of the two traits in question (e.g., male and female LRS). However, we did not estimate covariances between either among-individual variances or residual variances. Phenotypic variance (*V*_p_) was estimated as the differences between individuals in the focal trait, where *V*_a_, *V*_m_, V_i_ (if applicable), and *V*_r_ were summed to estimate *V*_p_. Heritabilities (*h*^2^), maternal effects (*m*^2^), and permanent environmental effects (pe^2^) were estimated as the proportion of phenotypic variance explained by the additive genetic variance, maternal variance, and individual variance, respectively. As all response variables followed a Poisson distribution, we report latent heritabilities, where the raw mean of a trait was added to the denominator of the heritability estimate (Nakagawa and Schielzeth 2010; Reid et al. 2011). This allowed for a heritability estimate, given fixed effects (Reid et al. 2011). Genetic correlations were estimated as the covariance between the additive genetic variances of each trait divided by the square root of the product of the variances of each trait (Lynch and Walsh 1998). Estimates of *h*^2^, *m*^2^, pe^2^, *V*_a_, *V*_m_, V_i_, and genetic correlations are reported with 95% credible intervals. The estimates reported in Tables 2 and 3 were all estimated from univariate models to measure the variance components of traits regardless of their genetic correlations with other traits.

**Table 2:** Estimates of heritability (*h*^2^), maternal effects (m^2^), and of variance components, including additive genetic (*V*_a_), maternal (*V*_m_), residual (*V*_r_), and credible intervals (CI) are reported for lifetime reproductive success (LRS) of North American red squirrels (*Tamiasciurus hudsonicus*). Additionally, we report the mean, standard deviation, median, and number of individuals measured for each fitness component

Fitness component	Mean ± SD	Median	*N*	*h*^2^	CI	*m*^2^	CI
LRS	1.1 ± 3.5	0	2981	4.90E−04	3.0E−08 to 0.07	0.07	0.02 to 0.14
Female LRS	1.4 ± 3.9	0	2133	6.80E−04	8.5E−11 to 0.10	0.08	0.01 to 0.14
Male LRS	0.3 ± 1.6	0	848	1.10E−03	7.1E−10 to 0.39	0.10	0.10 to 0.37
Mast LRS	1.5 ± 3.9	0	756	1.20E−03	1.8E−10 to 0.29	0.11	0.01 to 0.23
Nonmast LRS	1.0 ± 3.3	0	2225	1.60E−04	4.5E−13 to 0.06	0.12	0.04 to 0.21

Fitness component	*V*_a_	CI	*V*_m_	CI	*V*_r_	CI
LRS	4.00E−03	5.2E−07 to 1.1	1.2	3.4E−01 to 2.2	12.9	10.3 to 14.8
Female LRS	4.90E−03	1.3E−09 to 1.5	0.77	3.1E−01 to 2.2	11.9	9.7 to 14.8
Male LRS	1.70E−02	1.8E−08 to 6.7	2.4	1.5E−01 to 6.5	8.84	4.1 to 14.7
Mast LRS	1.20E−02	2.1E−09 to 2.8	0.94	1.1E−01 to 2.3	6.75	4.4 to 9.6
Nonmast LRS	5.10E−03	7.9E−12 to 1.2	1.98	6.3E−01 to 4.1	14.5	11.7 to 18.9

**Table 3:** Estimates of heritability (*h*^2^), maternal effects (*m*^2^), permanent environmental effect (pe^2^), and of variance components, including additive genetic (*V*_a_), maternal (*V*_m_), residual (*V*_r_), and credible intervals (CI) are reported for annual reproductive success (ARS) of North American red squirrels (*Tamiasciurus hudsonicus*). Additionally, we report the mean, standard deviation, median, and number of individuals measured for each fitness component

Fitness component	Mean ± SD	Median	*N*	*h*^2^	CI	*m*^2^	CI	pe^2^	CI
ARS	1.9 ± 2.1	2	1682	6.30E−04	6.6E−08 to 0.16	0.10	0.05 to 0.17	0.20	0.09 to 0.30
Female ARS	2.1 ± 2.2	2	1361	6.40E−04	1.8E−09 to 0.11	0.13	0.07 to 0.22	0.17	0.08 to 0.25
Male ARS	1 ± 1.8	0	321	4.10E−04	6.5E−10 to 0.15	0.09	0.03 to 0.24	0.09	0.03 to 0.29
Mast ARS	3.0 ± 3.0	3	286	1.20E−03	1.1E−07 to 0.20	0.17	0.06 to 0.31	0.21	0.08 to 0.46
Nonmast ARS	1.7 ± 1.8	1	1396	7.80E−04	1.7E−07 to 0.15	0.10	0.05 to 0.18	0.19	0.08 to 0.28

Fitness component	*V*_a_	CI	*V*_m_	CI	*V*i	CI	*V*_r_	CI
ARS	6.50E−04	9.0E−08 to 0.22	0.13	6.2E−02 to 0.20	0.26	0.1 to 0.4	0.37	0.3 to 0.5
Female ARS	7.10E−04	1.8E−09 to 0.11	0.12	6.2E−02 to 0.22	0.15	7.7E−02 to 0.26	0.26	0.2 to 0.4
Male ARS	1.20E−03	2.1E−09 to 0.51	0.29	7.8E−02 to 0.80	0.30	9.0E−02 to 0.99	1.40	0.9 to 2.3
Mast ARS	1.20E−03	1.5E−07 to 0.27	0.19	7.3E−02 to 0.40	0.28	9.1E−02 to 0.63	0.30	0.1 to 0.7
Nonmast ARS	1.30E−03	3.0E−07 to 0.19	0.13	6.1E−02 to 0.24	0.20	9.8E−02 to 0.36	0.37	0.3 to 0.5

We included fixed effects in our models to account for large differences between years in the availability of food resources (LaMontagne and Boutin 2007) and juvenile recruitment (McAdam and Boutin 2003) and did not include additional fixed effects that might have otherwise explained variation in fitness (Wilson 2008). We included a fixed effect for cohort in all models to account for effects of the environment in the year an individual was born, and we accounted for the effect of year in all annual models because environmental conditions can vary substantially among years (LaMontagne and Boutin 2007). We included year or cohort as a fixed effect rather than a random effect so that we could estimate the magnitude of other random effects marginal to the large annual changes in mean fitness. The inclusion of these fixed effects meant that fitness was measured relative to the mean fitness for that cohort or year, although this is not equal to relative fitness. As noted above, separate analyses based on relative fitness (not shown) did not affect our conclusions. The significance of fixed effects was estimated using generalized linear models with a quasipoisson error distribution (logit link function) in R. We used an *F*-test in our analysis of deviance (Venables and Ripley 2002). Estimates of population means are reported ± the standard deviation.

We used parameter expanded prior probabilities because we wanted to have noninformative priors (Hadfield 2010), which reflected previous ignorance toward our hypotheses (Ellison 2004). To ensure that each model properly converged, we used a Heidelberger and Welch convergence diagnostic (Heidelberger and Welch 1983) and a Geweke convergence diagnostic (Geweke 1992). We used more than one diagnostic because neither is considered to be reliable in all circumstances (Cowles and Carlin 1996). All models were converged as determined by both tests except where noted.

## Results

Mean lifetime reproductive success (LRS) was 1.1 ± 3.5 pups, with a mean female LRS of 1.4 ± 3.9 pups and a mean male LRS of 0.3 ± 1.6 pups. The mean annual reproductive success (ARS) of males and females together was 1.9 ± 2.1 pups, with a mean ARS of 3.0 ± 3.0 pups in mast years and a mean ARS of 1.7 ± 1.8 pups in nonmast years. Mean LRS of individuals averaged 1.5 ± 3.9 for individuals born in mast years and 1.0 ± 3.3 for individuals born in nonmast years. LRS differed significantly between cohorts (*F*_23,2957_ = 10.92, *P* < 0.0001), and ARS differed significantly between both cohorts (*F*_23,4575_ = 28.66, *P* < 0.0001) and years (*F*_23,4552_ = 57.37, *P* < 0.0001).

Direct genetic variation (*V*_a_) in LRS was very low and did not increase when considered separately as female LRS, male LRS (Table 2), and mast LRS or nonmast LRS (Table 2). The same was true for ARS (Table 2). Heritability of LRS was between 0.00016 and 0.0011, and heritability of ARS was between 0.00041 and 0.0012. As a result of these very low levels of *V*_a_, all estimated genetic correlations had very broad credible intervals and were essentially not estimable (Table 4).

**Table 4:** Genetic and maternal correlations between each of male and female fitness (both LRS and ARS) and mast and nonmast fitness (LRS and ARS) in North American red squirrels (*Tamiasciurus hudsonicus*). Ninety-five percentage of credible intervals (CI) broadly overlapped zero in all cases

Fitness component	Genetic correlation	CI	Maternal correlation	CI
LRS sexual antagonism	−0.95	−0.99 to 0.78	0.73	−0.20 to 0.99
LRS temporal fluctuations^1^	0.96	−0.84 to 0.99	−0.18	−0.91 to 0.72
ARS sexual antagonism	−0.85	−0.99 to 0.73	−0.83	−0.99 to 0.79
ARS temporal fluctuations	0.53	−0.71 to 0.99	0.28	−0.83 to 0.99

1 Bivariate animal model for LRS did not converge according to Heidelberger and Welch convergence test.

We found moderate maternal effects that were larger than additive genetic effects in all cases (Tables 2 and 3). However, we did not find evidence of maternal correlations between the sexes or between the environments, as all maternal correlations had very broad credible intervals and overlapped with zero (Table 4).

## Discussion

In order to evolve, a trait must be genetically correlated with fitness (Price 1970), which requires genetic variance in both the focal trait and fitness (Houle 1991). Here, we estimated a *V*_a_ of 0.004 and a heritability of 0.00049 for LRS. This suggests that while evolution in this system is possible, it would proceed very slowly. This amount of genetic variation in fitness corresponds to an evolvability of LRS equal to 0.0033 where evolvability is equal to the additive genetic variance divided by the squared mean of the trait (Hansen et al. 2011). This can be interpreted as the additional number of pups added to the mean per generation. This suggests that it would take around 275 generations for the average lifetime reproductive success to increase by one pup. This is in contrast to evolvability that can be estimated from the female red deer system of 0.20 relative calves per year (Foerster et al. 2007) or that from female collared flycatchers of 0.085 chicks per lifetime per generation (Merilä and Sheldon 2000), which predict, respectively, 0.63 generations and 5.3 generations to add one offspring to the mean.

Estimates of *V*_a_ for male and female fitness as well as fitness measured separately in mast and nonmast environments were also very small. MCMCglmm does not allow for negative estimates of variances (Hadfield 2010), which is why none of the 95% credible intervals for variance components overlapped with zero (Tables 2 and 3). Nevertheless, the modes of the posterior distributions that we estimated here were extremely low, and the power of our large pedigree allowed us to place fairly restricted upper bands on the 95% credible intervals, particularly for LRS itself (Table 2).

The low levels of genetic variation that we found prevented us from reasonably estimating genetic correlations between the sexes or between the environments. Genetic correlations require that both traits have significant genetic variation (Houle 1991). In our case, the low levels of *V*_a_ resulted in very broad credible intervals for our estimated genetic correlations, which impaired their ability to provide useful tests for fitness trade-offs between fitness components. Nevertheless, our estimates of very low levels of genetic variance in fitness and components of fitness that we thought might trade-off provide clear evidence against balancing selection as a mechanism maintaining direct genetic variance in fitness through sexual antagonism or fitness trade-offs between environments.

The low genetic variance in fitness that we have documented for red squirrels is consistent with theoretical predictions (Robertson 1955; Fisher 1958; Jones 1987) and several previous studies of genetic variance in fitness from the wild (Table 1), but is in contrast with others that have found moderate levels of genetic variance in fitness (Table 1). For example, Foerster et al. (2007) found heritabilities of 0.09 and 0.07 for female and male fitness, respectively. Evidence of high V_a_s for each of male and female fitness, however, is not necessarily indicative of the evolutionary potential of a population, as sexual antagonism can constrain the evolution of fitness. For example, the evolvability we calculated from the heritabilities of male and female fitness presented in red deer (Kruuk et al. 2000; Foerster et al. 2007) suggests the potential for very rapid microevolution, but this is unlikely to occur given the strong sexual antagonism found in red deer (Foerster et al. 2007), whose population has remained relatively stable (Kruuk et al. 2000). This example illustrates the importance of estimates of whole population heritabilities and evolvabilities of fitness in addition to the analysis of fitness components such as male and female fitness.

Maternal effects can be defined as the effects of a mother on the phenotypes of her offspring above and beyond her direct genetic contribution to offspring phenotype (Mousseau and Fox 1998). Maternal effects on fitness have been previously measured for both red deer and house sparrows, although in other species, maternal effects on fitness were found to be nonsignificant (Table 1). Our estimates of maternal effects are similar to the estimates of maternal effects of LRS in female red deer (0.16 ± 0.041; Kruuk et al. 2000), although they are lower than those estimated for house sparrows (0.33; Schroeder et al. 2011).

The maternal effects that we have demonstrated (Tables 2–4) could be very important to evolution in the red squirrel system if they are heritable, which could lead to unexpected patterns of evolution (Kirkpatrick and Lande 1989). Maternal genetic effects, where a mother's genes affect her offspring's phenotype, are a particular kind of maternal effect that we did not separate from maternal environmental effects. In addition to the high levels of maternal effects we have documented here, maternal genetic effects have been previously shown to make important contributions to variation in offspring growth rates in red squirrels (McAdam et al. 2002) and their evolution (McAdam and Boutin 2004). Maternal genetic effects have also been quantified in Soay sheep (*Ovis aries*) where birth weight, birth date, and natal litter size are highly correlated with fitness (Wilson et al. 2005). Future research in this system will include investigation of maternal genetic effects, as the additive genetic variance needed for short-term evolution in this system may reside in maternal genetic effects rather than direct genetic effects.
